# Pleomorphic adenoma of the epiglottis: A systematic review

**DOI:** 10.1097/MD.0000000000044541

**Published:** 2025-09-12

**Authors:** Nian Dong, Demin Li, Fan Yang, Yuhui Ren, Yungang Wu, Hui Zhang

**Affiliations:** a Clinical Medical University of Jining Medical University (Affiliated Hospital), Jining, Shandong, China; b Department of Otorhinolaryngology Head and Neck Surgery, Affiliated Hospital of Jining Medical University, Jining, Shandong, China.

**Keywords:** epiglottis, laryngeal, major salivary glands, pleomorphic adenoma

## Abstract

**Background:**

Pleomorphic adenoma (PA) predominantly occurs in major salivary glands, with epiglottic involvement being exceptionally rare. This study systematically reviews the epidemiological characteristics, clinical manifestations, diagnostic approaches, therapeutic interventions, and prognostic outcomes of PA of the epiglottis.

**Methods:**

Two independent investigators screened the literature and extracted data, including the following databases: PubMed, Web of Science, CAS SciFindern, EMBASE, Scopus, Cochrane Library databases, and regional databases including SciELO, Dimensions, and CNKI. In addition, we also searched grey literature sources, ClinicalTrials.gov, and PROSPERO. Furthermore, there are also Google Scholar and Baidu Scholar. The literature search was current through July 2025 and used a narrative synthesis method to comprehensively evaluate the case characteristics, clinical manifestations, imaging characteristics, treatment methods, and follow-up results.

**Results:**

A total of 28 patients with PA of the epiglottis were included. The mean age at diagnosis was 58.8 ± 14.3 years (range: 25–83 years), with the following age distribution: 25–35 years (n = 1, 3.6%), 36–45 years (n = 5, 17.9%), 46–55 years (n = 9, 32.1%), 56–65 years (n = 4, 14.3%), 66–75 years (n = 6, 21.4%), and 76–85 years (n = 3, 10.7%). The cohort comprised 19 males (67.9%) and 9 females (32.1%). The predominant clinical manifestations included pharyngeal foreign body sensation, dyspnea, and dysphagia. Tumors were localized to the laryngeal surface of the epiglottis in 13 cases (46.4%) and the tongue surface in 8 cases (28.6%). Tumor dimensions ranged from a maximum of 6.0 × 5.0 cm to a minimum of 1.3 × 1.0 cm. A complete capsule was identified in 13 cases (46.4%), while absence of an intact capsule was noted in 1 case (3.6%); capsule status was not documented in the remaining 14 cases (n = 50.0%). Twenty-seven patients underwent surgical resection, including open surgery and other operative methods; open surgery was performed in 14 cases (50%). No instances of recurrence, malignant transformation, or distant metastasis were observed during follow-up.

**Conclusion:**

This study represents the first systematic review to comprehensively evaluate the characteristics of PA of the epiglottis. The patient exhibits no typical laryngeal symptoms, and surgical resection serves as the primary therapeutic approach. Given the potential for recurrence and malignant transformation, long-term postoperative follow-up is required.

## 1. Introduction

Pleomorphic adenoma (PA), also termed mixed tumor,^[1]^ is the most common benign neoplasm of salivary gland origin.^[2]^ It predominantly arises in major salivary glands, with approximately 80% occurring in the parotid glands, while minor salivary glands account for about 10% of cases.^[3–5]^ Epidemiologically, PA is most frequently diagnosed in individuals aged 40 to 60 years, with a female predominance.^[5–7]^ The etiology remains unclear, and these lesions typically exhibit indolent growth with nonspecific symptoms in early stages.^[8]^ Histologically, PA is characterized by a circumscribed proliferation of epithelial or myoepithelial cells demonstrating morphological diversity, including spindle cells, cuboidal cells, plasmacytoid cells, squamous cells, and mucinous cells. The stromal components contain mucin deposits, myxoid tissue, and chondroid matrix.^[5,8–10]^ Diagnostic imaging modalities such as ultrasonography, computed tomography (CT), and magnetic resonance imaging (MRI) may aid localization, but definitive diagnosis requires histopathological examination. Surgical resection remains the primary therapeutic approach.^[10,11]^ Reported recurrence rates range from 0.5% to 10%, with malignant transformation occurring in approximately 6% of cases.^[12–16]^ Epiglottic involvement by PA is exceptionally rare, and no standardized diagnostic and therapeutic protocols exist globally for this anatomic variant. Current literature predominantly consists of isolated case reports, highlighting a critical gap in systematic evidence synthesis. This study therefore aims to systematically review published cases of epiglottic PA, consolidating data on clinical characteristics, radiological findings, management strategies, and long-term outcomes to inform evidence-based clinical decision-making.

## 2. Materials and methods

### 2.1. Databases and search strategies

A comprehensive literature search was performed in PubMed, Web of Science, CAS SciFindern, Embase, Scopus, Cochrane Library databases, and regional databases including SciELO, Dimensions, and CNKI. In addition, we also searched gray literature sources, ClinicalTrials.gov and PROSPERO. Furthermore, there are also Google Scholar and Baidu Scholar databases in July 2025. The search strategy utilized the following Boolean syntax: ((“Compound adenoma” OR “Pleomorphic adenoma” OR “mixed tumor”) AND (“epiglottis” OR “laryngeal operculum” OR “epiglottic” OR “epiglot*”)) combined with ((“larynx” OR “laryngeal” OR “throat” OR “pharynx” OR “maw” OR “guttur” OR “gullet” OR “gula” OR “laryngopharynx”) AND (“Compound adenoma” OR “Pleomorphic adenoma” OR “mixed tumor”)). The initial search yielded 443 citations in PubMed, 162 in Web of Science, 212 in CAS SciFindern, 884 citations in Embase, 4352 citations in Scopus, 69 citations in Cochrane Library, 159 citations in SciELO, 223 citations in Dimensions, 128 citations in CNKI, 993 citations in Google Scholar, and 1797 citations in Baidu Scholar. No available data were found in the gray literature, ClinicalTrials.gov or PROSPERO. Following rigorous title/abstract screening and full-text evaluation, 29 articles met the predefined inclusion criteria and were included in this systematic review.

### 2.2. Inclusion and exclusion criteria

#### 2.2.1. Inclusion criteria

1. Patients with histopathologically or radiologically confirmed PA of the epiglottis.
2. No restrictions on publication date.
3. Study types: case reports, systematic reviews, or original studies.

#### 2.2.2. Exclusion criteria

1. Malignant neoplasms or borderline tumors with malignant potential.
2. An article with missing basic information.

### 2.3. Data collation and risk of bias

This article was methodologically assessed following the Preferred Reporting Items for Systematic Reviews and Meta-Analyses 2020 checklist. Following the application of predefined inclusion and exclusion criteria, all eligible articles underwent rigorous title/abstract screening and full-text evaluation, including manual review of reference lists. The final included literature exclusively comprised case reports. Data extraction was performed using a standardized template capturing: author(s), age, gender, symptoms, tumor localization, size, tumor dimensions capsule status, therapeutic intervention, and follow-up outcomes.

Two investigators independently performed data abstraction. Challenges in data retrieval from older publications were systematically documented. Any discrepancies in data interpretation between the 2 primary investigators were adjudicated by a third senior researcher through consensus-building review of source materials.

### 2.4. Statistical analysis

Given the limited sample size across included studies, this analysis did not conduct intergroup comparisons or multivariate analyses. Descriptive statistical analyses were primarily employed to aggregate data on patient demographics, clinical presentations, imaging features, therapeutic approaches, and follow-up outcomes.

Categorical variables (e.g., gender, symptom profiles) were expressed as frequencies and percentages, while continuous variables (e.g., age) were summarized using mean values. The results were systematically presented through tabular or graphical formats to enhance interpretability and visual clarity.

## 3. Results

**Figure 1.**
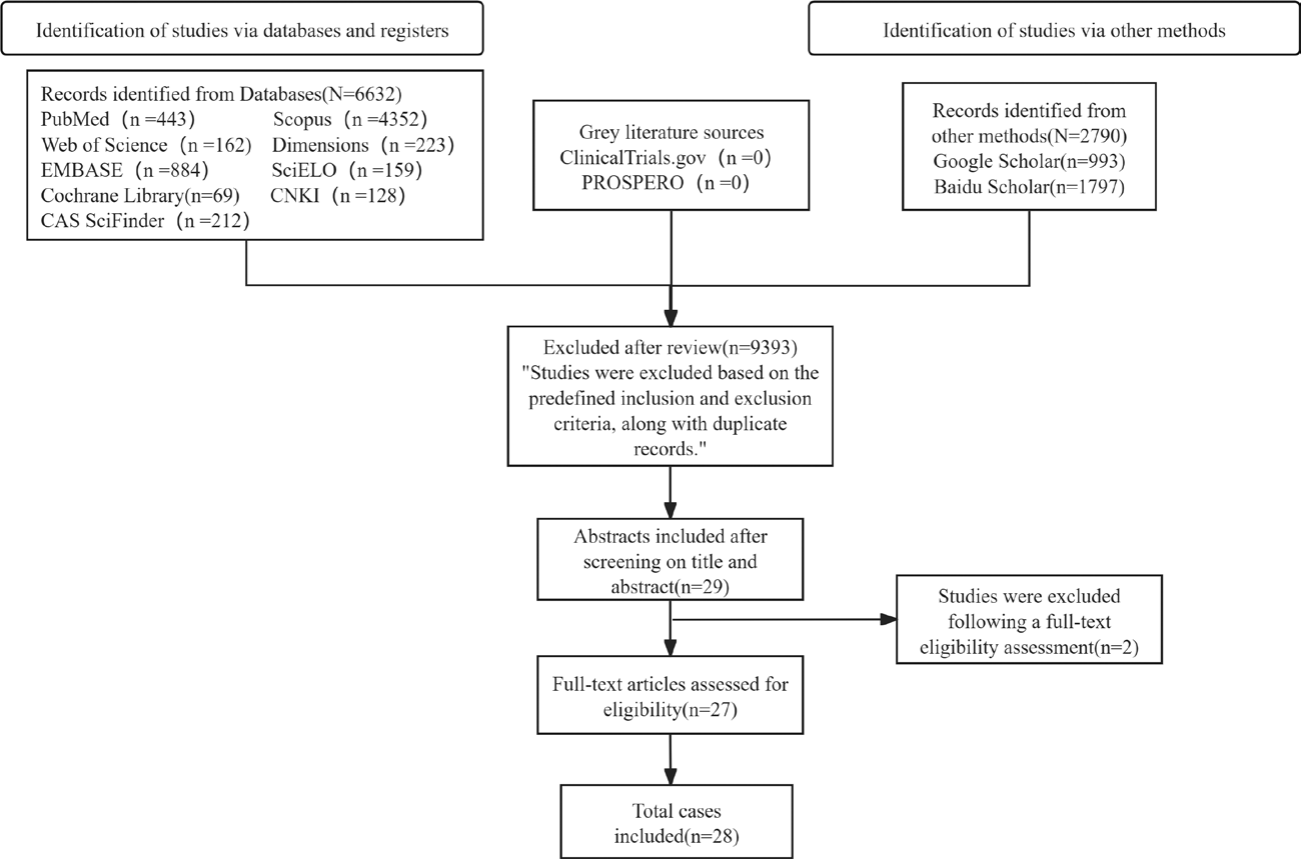
Flow diagram of database and registry searches.

A total of 29 articles were initially included based on predefined eligibility criteria. However, 2 articles were excluded due to incomplete demographic data, resulting in 28 analyzable cases of epiglottic PA (Table 1; Fig. 1). The mean age at diagnosis was 58.8 ± 14.3 years (range: 25–83), with the following age distribution: 25 to 35 years (n = 1, 3.6%), 36 to 45 years (n = 5, 17.9%), 46 to 55 years (n = 9, 32.1%), 56 to 65 years (n = 4, 14.3%), 66 to 75 years (n = 6, 21.4%), and 76 to 85 years (n = 3, 10.7%). The cohort comprised 19 males (67.9%) and 9 females (32.1%) (Table 2).

**Table 1:** Characteristics of included studies.

References	Age (yr) Sex (M: Male,F: Female)	Symptom(s)	Location	Size	Encapsulation	Therapy	Follow-up
Stawiński^[17]^	39 M	Dyspnea, dysphagia	–	6.0 × 5.0 cm	–	Excision by pharyngotomy	–
Berger et al^[18]^	64 F	Suffocation, hoarsenes, dysphagia, weight loss	–	–	–	Horizontal partial laryngectomy	No recurrence
Jokinen et al^[19]^	61 F	–	Laryngeal surface	–	Yes	Excision by pharyngotomy	No recurrence at 4 mo
Vilde et al^[20]^	25 M	Dysphonia, dysphagia	Tongue surface	5.0 × 4.0 × 3.0 cm	–	Epiglottectomy	–
Cotelingam et al^[21]^	69 M	Lumpy sensation	Laryngeal surface	3.7 × 3.5 × 2.1 cm	Yes	Excision partial epiglottis and lump by pharyngotomy	No recurrence at 15 mo
Tobin^[22]^	70 M	Hoarseness, difficulty swallowing	Laryngeal surface	4.0 cm, diameter	Yes	Excision by pharyngotomy	Died of other diseases after 7 wk
Baptista et al^[23]^	42 M	Dysphagia, dysphonia	Tongue surface	2.5 cm, diameter	–	Excision partial epiglottis and lump by pharyngotomy	No recurrence
Suttner et al^[24]^	82 M	–	Tongue surface	2.2 × 1.8 × 1.5 cm	–	Excision partial epiglottis and lump by CO_2_ laser	No recurrence at 1 yr
Ito et al^[25]^	79 F	Lumpy sensation, dysphagia	Laryngeal surface	2.0 × 1.5 cm	–	Excision by Nd-YAG laser	No recurrence at 5 mo
Dubey et al^[26]^	45 F	Muffled voice and food particles sticking in the throat	Laryngeal surface	4.0 × 3.0 cm	Yes	Excision epiglottis by pharyngotomy	No recurrence at 4 yr
Dai et al^[27]^	49 M	Discomfort when swallowing	Laryngeal surface	2.0 cm, diameter	No	Excision by laryngoscope supporting	–
Alzate Amaya et al^[28]^	66 F	–	Laryngeal surface	1.3 × 1.1 cm	–	Excision by CO_2_ laser	–
Weng et al^[29]^	43 M	Hoarseness, lumpy sensation	Laryngeal surface	1.5 cm, diameter	Yes	Excision by laryngoscope supporting	–
Terazono et al^[30]^1	71 F	Pharyngalgia, hoarseness	Laryngeal surface	1.5 cm, diameter	Yes	Surgical resection	No recurrence at 1 yr
Matsumoto^[31]^	68 M	Foreign-body sensation, unclear voice	–	–	–	Surgical resection	No recurrence at 3 yr
Suzuki et al^[32]^	47 M	Pharyngalgia, dysphagia, dyspnea	Tongue surface	4.0 cm, diameter	Yes	Excision by horizontal laryngectomy	–
Ohta et al^[33]^	46 M	Cough	Tongue surface	5.0 × 4.0 × 3.0 cm	Yes	Surgical resection	–
Peng et al^[34]^	51 F	Foreign-body sensation	Laryngeal surface	2.0 × 1.5 × 1.0 cm	Yes	Suprahyoid epiglottis tumor resection	No recurrence at 2 yr
Ling et al^[35]^	60 M	Lumpy sensation	Tongue surface	4.0 × 2.0 × 2.0 cm	Yes	Excision by pharyngotomy	No recurrence at 3 yr
Ohkabo et al^[36]^	51 M	Foreign-body sensation	Laryngeal surface	1.3 × 1.3 × 1.2 cm	–	Epiglottectomy under laser with Davis gag exposure	–
Morita et al^[37]^	73 M	Foreign-body sensation	Laryngeal surface	2.5 × 2.5 cm	–	Excision by pharyngotomy	–
Zhang and Ma^[38]^	55 M	Pharyngalgia	Tongue surface	2.0 × 2.0 × 1.0 cm	Yes	Open neck surgery	No recurrence at 2 yr
Rong^[39]^	42 M	Hoarseness, foreign-body sensation	–	1.5 × 1.0 × 1.0 cm	Yes	Surgical resection	No recurrence at 2 yr
	50 M	Hoarseness, obstructive dysphagia	–	2.2 × 2.0 × 1.0 cm	Yes	Excision partial epiglottis and lump	No recurrence at 2 yr
Sun and Peng^[40]^	83 F	Dysphagia, foreign-body sensation	Tongue surface	4.0 × 3.0 × 3.0 cm	–	Excision by laryngoscope supporting	No recurrence at 3 mo
Li et al^[41]^	48 F	Hoarseness, obstructive dysphagia	Laryngeal surface	3.5 × 3.0 × 1.5 cm	–	Suprahyoid epiglottis tumor resection	No recurrence at 6 mo
Cheng and Chen^[42]^	60 M	Pharyngalgia, dysphagia	–	3.0 × 3.0 × 2.0 cm	–	Excision by pharyngotomy	No recurrence at 1 yr
Nagashima et al^[43]^	52 M	Neck swelling	–	2.5 × 2.0 × 2.0 cm	–	–	–

**Table 2:** Age and gender characteristics of pleomorphic adenoma of the epiglottis.

Variables	No.	%
Age (yr)		
25–35	1	3.6
36–45	5	17.9
46–55	9	32.1
56–65	4	14.3
66–75	6	21.4
76–85	3	10.7
Gender (male, female)		
Male	19	67.9
Female	9	32.1

The most prevalent symptoms were pharyngeal foreign body sensation, dysphagia, dysphonia, and pharyngalgia. Tumor localization was unspecified in 7 cases. Among documented cases, the laryngeal surface of the epiglottis was more frequently involved (n = 13, 46.4%) compared to the lingual surface (n = 8, 28.6%) (Table 3). Tumor dimensions ranged from 1.3 × 1.1 cm (minimum) to 6.0 × 5.0 cm (maximum). Histopathological evaluation revealed complete encapsulation in 13 cases (46.4%), unenveloped in 1 case (3.6%), and undocumented capsule status in the remaining 14 cases (50.0%).

**Table 3:** Presenting symptoms and location of patients with pleomorphic adenoma of the epiglottis.

Variables	No.	%
Presenting symptoms		
Globus pharyngeus/dysphagia	22	78.6
Dysphonia	10	35.7
Pharyngalgia	3	10.7
Dyspnea	3	10.7
Weight loss/cough/neck swelling	3	10.7
Location		
Laryngeal surface	13	46.4
Tongue surface	8	28.6

Twenty-seven patients underwent surgical resection, including open surgery and other operative methods, and open surgery was performed in 14 cases (50%). No instances of recurrence, malignant transformation, or metastatic spread were reported during follow-up periods.

## 4. Discussion

### 4.1. Fundamental characteristics of PA of the epiglottis

PA, first described by Willis,^[6]^ is a common benign mixed tumor predominantly arising in major salivary glands, particularly the parotid gland. Ectopic occurrences are rare but have been documented in diverse anatomical sites including the mandible, external auditory canal, lacrimal gland, nasal septum, breast, lung, esophagus, paranasal sinuses, skull base, and trachea.^[4]^ Within the larynx, PA primarily involves the supraglottic region, with the epiglottis being the most frequently affected site.^[4,44,45]^ Notably, Jones et al proposed that seromucous glands in the nasal cavity, larynx, and bronchi (though non-salivary in function) exhibit histological similarity to minor salivary glands,^[46]^ rendering their susceptibility to PA development mechanistically plausible.

Prior investigations into epiglottic PA have been limited, with no systematic reviews identified to date. Through comprehensive literature synthesis, this study aims to provide an integrative analysis of the disease, offering enhanced clinical insights for evidence-based management.

Among analyzed cases (mean age at diagnosis: 58.8 ± 14.3 years), the majority (36–75 years) demonstrated a male predominance (male-to-female ratio is approximately 2:1) and preferential involvement of the laryngeal epiglottic surface over the lingual surface, consistent with earlier reports by Dubey et al.^[26]^ The largest documented tumor measured 6.0 × 5.0 cm.^[47]^ Characteristically, these lesions present as slow-growing, painless, solitary masses with smooth surfaces. The exact pathogenesis of epiglottic PA remains elusive. Given its histopathological congruence with salivary gland PA, etiological parallels may be drawn. Established risk factors for salivary PA include: genetic predisposition, environmental exposures (radiation, tobacco, SV40 polyomavirus), and occupational chemical contact. Notably, while smoking has not been definitively linked to PA tumorigenesis, its role in promoting neoplastic progression warrants further investigation.^[5,6,47–49]^

Nonspecific symptoms predominated in reviewed cases: pharyngeal foreign body sensation, dysphagia, dyspnea, pharyngeal discomfort, hoarseness, etc and even weight loss. There are also patients who do not have any clinical manifestations and have the mass inadvertently discovered, such as during endotracheal intubation.^[26,17–25,27–36,38–42,50]^

### 4.2. Diagnostic imaging

PA of the epiglottis is typically identified through clinical evaluation and indirect laryngoscopy. Advancements in video laryngoscopy have enabled direct visualization of tumor surface characteristics, primary anatomical origin, and the degree of glottic obstruction. Similar to other laryngeal neoplasms, conventional imaging modalities (e.g., contrast-enhanced CT, MRI) are routinely employed to complement pathological evaluation and guide therapeutic planning.

Previous studies have demonstrated that ultrasound has a limited role in the diagnosis and treatment of epiglottic PA, therefore it will not be discussed in detail in this study. Positron emission tomography-computed tomography is primarily utilized for assessing systemic metastasis in malignant tumors. Although epiglottic PA carries malignant transformation potential, no clinical applications of positron emission tomography-computed tomography in this specific context have been documented to date.

Fine-needle aspiration cytology is widely used in preoperative evaluation of salivary gland tumors, particularly parotid PAs.^[51,52]^ Studies have demonstrated that its sensitivity for distinguishing benign from malignant parotid tumors ranges from 71% to 100%, with specificity values between 33% and 100%. The positive predictive value, negative predictive value, and accuracy vary widely, ranging from 33% to over 80%.^[53]^ Despite these limitations, fine-needle aspiration cytology remains clinically valuable in the management of salivary gland tumors.^[54]^ However, its preoperative application in epiglottic PA is significantly limited and therefore will not be discussed in detail.

Based on literature review, radiography provides more detailed localization information compared to visual inspection, with characteristic findings including: smooth-surfaced, well-defined soft tissue density masses^[21]^; homogeneous dense opacities.^[26]^

CT and MRI are extensively utilized in the evaluation of epiglottic PAs, serving not only for initial characterization but also for assessing the extent of local invasion. In the study by Dai et al,^[27]^ CT features demonstrated irregularly marginated, lobulated masses with heterogeneous enhancement. MRI findings revealed solitary nodular lesions exhibiting moderate heterogeneous enhancement and ill-defined borders, making differentiation between benign and malignant entities challenging. Characteristic signal patterns included hyperintensity on T2-weighted imaging (T2WI), hypointensity on T1-weighted imaging (T1WI), and signal reduction on apparent diffusion coefficient (ADC) maps.^[25]^ However, comprehensive descriptions of CT/MRI manifestations specific to epiglottic PAs remain scarce in current literature.

Notably, in 2024, Furukawa et al reported the MRI manifestations of a subglottic PA. CT imaging revealed a well-defined mass with contrast enhancement. MRI demonstrated homogeneous isointensity on T1WI, heterogeneous hyperintensity on T2WI, mildly hyperintense signal on diffusion-weighted imaging, and normal to mildly elevated ADC values.^[55]^ In contrast, parotid PAs typically exhibit iso- to hypointensity on T1WI, hyperintensity on T2WI, and elevated ADC signals.^[55–57]^ These observations underscore the lack of consistency in imaging profiles between PAs occurring at different anatomical sites.

Given the substantial inter-site variability in radiological presentations, CT and MRI currently lack definitive diagnostic value for epiglottic PAs.

### 4.3. Histology

Microscopically, the neoplasm demonstrates proliferation of epithelial, myoepithelial, and stromal components. Histomorphological diversity constitutes the pathognomonic hallmark of PA, manifesting as: epithelial architecture: nested, trabecular, acinous, or tubular arrangements; stromal matrix: admixture of fibrous tissue, myxoid stroma, and pseudochondroid differentiation.

Notably, focal cytological atypia with low proliferative activity may be observed. In the study by Dai et al, immunohistochemical profiling revealed dual positivity for CK7 and p63.^[7,26,21,25]^ However, no established immunophenotypic profile exists for epiglottic PAs, as current literature remains limited to isolated case reports.

### 4.4. Differential diagnosis

PA of the epiglottis should be differentiated from other benign epiglottic lesions, such as epiglottic cysts and papillomas.

Epiglottic cysts typically arise from chronic mucosal inflammation of the epiglottis, leading to obstruction of the mucous gland ducts and subsequent formation of cystic masses. These cysts are commonly located in the vallecula, lingual surface, or free margin of the epiglottis, presenting as smooth, round, or hemispherical lesions. Large epiglottic cysts can cause severe dyspnea and, if infected, may progress to epiglottitis or abscess formation, posing a lethal risk. Thus, early diagnosis and surgical intervention are critical. Histopathologically, the cyst lining is usually composed of squamous and/or columnar epithelium.^[58–60]^ PAs of the epiglottis may grossly resemble epiglottic cysts, potentially leading to misdiagnosis. Therefore, meticulous clinical, imaging, and histopathological differentiation is essential during evaluation.

Papillomas are common benign tumors of the larynx, typically associated with human papillomavirus infection, and may also occur in the epiglottis. Tresley et al provided a detailed description of epiglottic papillomas, which grossly appear as pedunculated papillary growths. Histopathologically, they exhibit fibrovascular cores covered by mature keratinized stratified squamous epithelium, with no evidence of dysplasia or local tissue invasion. Primary treatment involves surgical excision. To minimize recurrence, narrow-margin resection at the lesion periphery is recommended.^[61–63]^ Chau et al reported a rare case of a collision tumor in the epiglottis, comprising both PA and squamous cell carcinoma. Definitive diagnosis relies on histopathological examination.^[44]^

### 4.5. Treatment

The primary treatment for PA of the epiglottis is surgical resection, with the surgical approach and extent determined by the tumor’s location and size.^[26]^ Traditional management predominantly involves open surgery through a transpharyngeal approach under general anesthesia. Ito et al have proposed that laser excision offers advantages such as preventing tumor cell dissemination, reducing intraoperative bleeding, minimizing postoperative edema, and decreasing scar formation.^[25]^ Currently, tumor resection under general anesthesia via suspension laryngoscopy has also become a viable option.

While there is no standardized treatment protocol for epiglottic PA, complete resection of the tumor along with its capsule is recognized as crucial. However, this conclusion does not appear to be definitively established, and it aligns with the standard surgical management principles for tumors. No conclusive research evidence supports the prognostic benefits of extended resection or adjuvant chemoradiotherapy. Studies indicate that postoperative radiotherapy may contribute to reduced recurrence rates, particularly demonstrating certain therapeutic efficacy in cases of recurrent PA.^[13,26]^

### 4.6. Recurrence or malignant transformation

Malignant PAs primarily include carcinoma ex pleomorphic adenoma (CXPA), carcinosarcoma, and metastasizing PA, with CXPA being the most common subtype. Approximately 6% of parotid gland PAs may undergo malignant transformation into CXPA.^[64,65]^ The precise pathogenesis remains unclear. Macroscopically, the tumors typically appear yellowish-white or grayish-white with a smooth or nodular surface. Histopathological examination reveals characteristic PA architecture, transitional zones, and carcinomatous areas. Treatment strategies are tailored to the lesion’s anatomical site, with lymph node dissection recommended if nodal metastasis is present.^[4,66]^

In 1989, Milford et al first reported a case of CXPA originating in the epiglottis. The patient underwent horizontal partial laryngectomy with functional neck dissection and remained recurrence-free at 1-year follow-up,^[67]^ while major salivary gland PAs exhibit notable recurrence rates,^[15,16]^ primarily due to incomplete capsular excision, Although Rooker and Nicholas et al suggested a potential association between incomplete capsular excision and tumor recurrence,^[12,13]^ over 50% of the included cases lacked documentation of capsular status in our analysis. The available data are insufficient to definitively support this conclusion, highlighting the need for future studies with comprehensive clinicopathological documentation and long-term follow-up to validate this hypothesis. This gap in evidence also reflects a current limitation in the diagnosis and management of epiglottic PA. None of the epiglottic PAs included in this study demonstrated recurrence during the follow-up period.

## 5. Conclusion

The principal limitations of this study include a limited sample size, an extended inclusion period, incomplete data collection in some cases, and despite expanding our search to include multiple databases and gray literature sources, the inherent limitations of systematic reviewing mean we cannot guarantee absolute completeness.

Furthermore, current evidence remains limited but suggests that complete excision may effectively prevent recurrence and malignant transformation; no robust clinical data are available to validate this perspective; it is necessary to conduct further research with larger-scale and more complete data to obtain more reliable evidence. However, to our knowledge, this represents the first systematic review addressing epiglottic PA and provides a comprehensive analysis of multiple facets of this rare disease.

Epiglottic PA demonstrates nonspecific clinical presentations, predominantly affecting middle-aged and elderly patients. Diagnosis requires a multidisciplinary approach incorporating clinical evaluation and imaging studies for preliminary assessment, with definitive confirmation relying on histopathological examination. Surgical resection constitutes the mainstay of treatment. Given the inherent risks of recurrence and malignant transformation, long-term postoperative surveillance should be implemented.

## Acknowledgments

All the authors thank Jining Medical University and its Affiliated Hospital for providing the learning platform and also express our gratitude to the corresponding authors for the careful guidance on this report.

## Author contributions

**Data curation:** Nian Dong, Fan Yang, Yuhui Ren.

**Formal analysis:** Nian Dong, Demin Li, Fan Yang, Yuhui Ren.

**Funding acquisition:** Hui Zhang.

**Investigation:** Nian Dong, Fan Yang, Yuhui Ren.

**Supervision:** Yungang Wu, Hui Zhang.

**Validation:** Yungang Wu, Hui Zhang.

**Writing – original draft:** Nian Dong, Demin Li.

**Writing – review & editing:** Yungang Wu, Hui Zhang.
